# Engineered Nanodelivery Systems to Improve DNA Vaccine Technologies

**DOI:** 10.3390/pharmaceutics12010030

**Published:** 2020-01-01

**Authors:** Michael Lim, Abu Zayed Md Badruddoza, Jannatul Firdous, Mohammad Azad, Adnan Mannan, Taslim Ahmed Al-Hilal, Chong-Su Cho, Mohammad Ariful Islam

**Affiliations:** 1 Nanotechnology Engineering Program, University of Waterloo, Waterloo, ON N2L 3G1, Canada; Michael.s.lim@edu.uwaterloo.ca; 2 Department of Chemical and Life Sciences Engineering, Virginia Commonwealth University, Richmond, VA 23284, USA; azmbadruddoza@vcu.edu; 3 Department of Medicine, Brigham and Women’s Hospital, Harvard Medical School, Boston, MA 02115, USA; jfirdous386@gmail.com; 4 Department of Chemical, Biological and Bioengineering, North Carolina A&T State University, Greensboro, NC 27411, USA; maazad@ncat.edu; 5 Department of Genetic Engineering and Biotechnology, Faculty of Biological Sciences, University of Chittagong, Chittagong 4331, Bangladesh; adnan.mannan@cu.ac.bd; 6 Department of Pharmaceutical Sciences, University of Texas El Paso, El Paso, TX 79968, USA; taalhilal@utep.edu; 7 Department of Agricultural Biotechnology and Research Institute of Agriculture and Technology, Seoul National University, Gwanak-gu, Seoul 08826, Korea; 8 Immuno-Oncology Division, Immunomic Therapeutics, Inc., Rockville, MD 20850, USA

**Keywords:** vaccine, nanoparticle, nanotechnology, adjuvant, immune cell targeting, peptide vaccine, DNA vaccine

## Abstract

DNA vaccines offer a flexible and versatile platform to treat innumerable diseases due to the ease of manipulating vaccine targets simply by altering the gene sequences encoded in the plasmid DNA delivered. The DNA vaccines elicit potent humoral and cell-mediated responses and provide a promising method for treating rapidly mutating and evasive diseases such as cancer and human immunodeficiency viruses. Although this vaccine technology has been available for decades, there is no DNA vaccine that has been used in bed-side application to date. The main challenge that hinders the progress of DNA vaccines and limits their clinical application is the delivery hurdles to targeted immune cells, which obstructs the stimulation of robust antigen-specific immune responses in humans. In this updated review, we discuss various nanodelivery systems that improve DNA vaccine technologies to enhance the immunological response against target diseases. We also provide possible perspectives on how we can bring this exciting vaccine technology to bedside applications.

## 1. Introduction

The development of new vaccines to address emerging diseases continues to be of great importance and a major focus of medical research. Traditional vaccines which include live-attenuated, inactivated, and subunit vaccines have historically been effective in treating infectious diseases [1]. However, vaccination against many rapidly evolving and emergent diseases such as cancers, HIV, and other similar infectious or virus-mediated diseases has not yet been successful [2]. These types of diseases require the induction of potent cell-mediated immune responses to eliminate intracellular pathogens and kill infected cells. In this regard, conventional protein-based vaccines are limited because they produce humoral immunity with little or no induction of cell-mediated immunity [3]. Moreover, each class of conventional vaccine technologies has detrimental disadvantages. For example, live-attenuated vaccines run the risk of reverting to their virulent state, while inactivated and subunit vaccines are often impotent, stimulating suboptimal immune responses that are incapable of generating protective immunity or therapeutic effects against many diseases [4].

DNA vaccine technologies present a promising alternative vaccine platform that has the potential to treat many diseases, such as cancers, atherosclerosis, and diabetes [2]. DNA vaccines function by encoding protein antigens into DNA, which are then delivered to cells for the production of specific proteins. Antigen-presenting cells (APCs) process these proteins and present them to lymphocytes that are capable of killing pathogens and infected cells. These antigens stimulate an immune response mimicking live infections and can induce both antigen-specific humoral and cell-mediated immunity [5]. DNA vaccine targets can be easily modified by changing their DNA sequences. The synthesis of plasmid DNA caused by the use of bacteria is very swift and inexpensive. Thus, DNA vaccine technology has great potential for future applications in the development of new vaccines for emerging and epidemic outbreaks.

Despite years of research, DNA vaccine technologies have had limited success in the clinic and none have been approved for human use. The weak immune response associated with low gene transfection of DNA vaccines is one of the major associated challenges [6]. Thus, the development of nanomaterial-based delivery systems to enhance the transfection efficiency and immunogenicity of DNA vaccines holds great promise. This review describes the progress of nanodelivery platform systems to improve the efficiency of DNA vaccine technologies and provides future perspectives on their potential for translation into clinical settings.

## 2. Challenges Associated with DNA Vaccines

**Figure 1:**
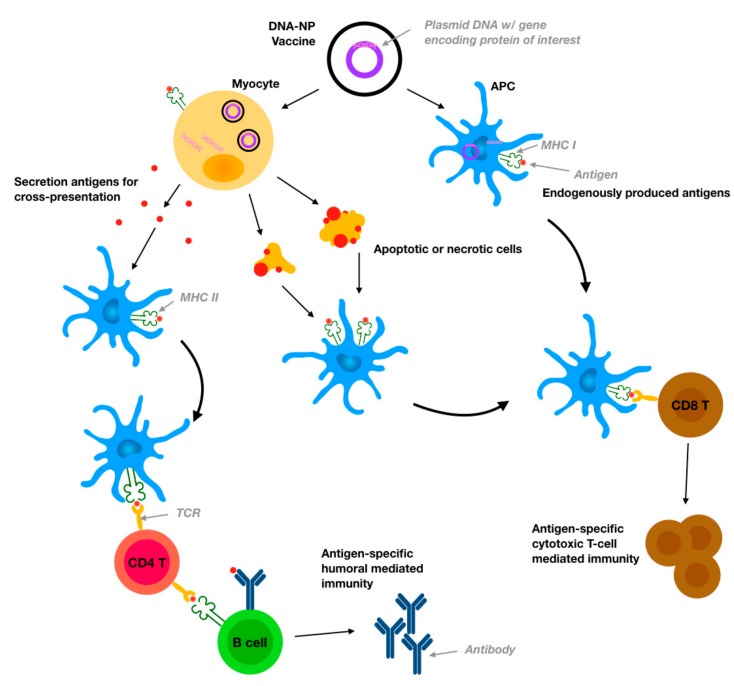
DNA vaccine mechanism for induction of cell-mediated and humoral immunity. The DNA-Nanoparticles (NPs) vaccine is administered through one of many routes, such as intramuscular, intravenous, intradermal, subcutaneous, intraperitoneal or oral, and transfects target cells, delivering the DNA payload to the cytosol. The DNA molecules translocate across the nuclear membrane, initiating gene transcription and subsequent synthesis of the encoded proteins. Antigen-presenting cells (APCs) are of primary importance for processing and presenting antigens to lymphocytes for the induction of robust immune responses. Specifically, APCs present antigens on major histocompatibility complex (MHC) I molecules either following direct transfection of the DNA vaccine or through cross-presentation from other cells, such as APC phagocytosis of transfected apoptotic or necrotic bodies. APCs also present antigens on MHC II molecules when transfected cells secrete the encoded proteins which are then endocytosed by APCs. APCs then can activate naive T cells by presenting antigens through the use of MHC I and T cell receptors (TCR), stimulating antigen-specific T-cell immune responses. Alternatively, through the MHC II pathway, processed antigens are presented to CD4 T cells, which mediate the induction of B cell responses that are capable of stimulating antigen-specific antibody-mediated immunity. Adapted from [7], which is an Open Access article distributed under the terms of the Creative Commons Attribution-NonCommercial-NoDerivatives License (http://creativecommons.org/licenses/by-nc-nd/4.0/)

In order to achieve successful gene transfection and stimulate the production of exogenous proteins, DNA vaccines must overcome a large number of extracellular and intracellular barriers. For intravenous delivery, DNA vaccines must be protected from degradation by DNase and phagocytic elimination through the reticuloendothelial system (RES) [7]. Furthermore, DNA vaccines must overcome inactivation through non-specific interactions with other proteins. Similarly, for intradermal, subcutaneous, and intraperitoneal vaccination, the DNA vaccine must be protected from degradation and elimination, and also reach targeted-immune cells for effective immune response stimulation. For oral delivery, the most critical challenges include resistance to the harsh gastric conditions in the digestive tract, as well as the protective mucosal membrane that prevents foreign pathogens and particulates from entering the body. At the cellular level, the physically large DNA molecule must cross the phospholipid cell membrane, which is negatively charged and inherently repulsive to the negative charge of the DNA structure. Once inside the cell, the DNA must escape the endosome or lysosome which engulfed the vaccine in order to reach the cytoplasm of the cell [8,9]. The DNA vaccine must then also remain intact against cytosolic nucleases and successfully translocate across the nuclear envelope for protein production to begin [10]. Specifically for DNA vaccination, it is ideal to target APCs in order to accelerate antigen presentation and stimulate robust cell-mediated immune responses. With the use of naked DNA, there is no reliable method to specifically target APCs, which is an additional drawback. Figure 1 broadly outlines the mechanism through which DNA vaccines induce antigen-specific immunity, and make apparent the need for effective delivery to APCs. All of the previously mentioned barriers to DNA delivery result in reduced gene transfection and ultimately low therapeutic efficacy.

## 3. Methods to Address Challenges of DNA Vaccine Delivery

Several technologies exist that can facilitate the delivery of plasmid DNA to cells to potentiate immune responses. Electroporation is one such technology that involves delivering electrical pulses to induce temporary and reversible permeability of the cell membranes. This, in turn, facilitates transient and stable cellular entry of DNA molecules [11]. Although electroporation is easy and rapid, enabling the transfection of many cells in a short period of time, the high voltage applied can cause significant cell death and physical pain to the patient. Furthermore, stimulated permeabilization can result in the nonspecific transport of molecules across cellular membranes [12]. Other mechanically facilitated DNA delivery methods include needle-free pneumatics or jet injectors which function by utilizing a pressurized chamber to forcefully inject a plasmid DNA containing solution into skin or muscle tissue [13]. Needle-free technology not only enables highly efficient and painless drug delivery, it also removes the complications associated with needles including needle stick injury and needle reuse. The disadvantages of needle free-technology are that they are generally expensive and sophisticated devices that require training to administer and are not compatible with intravenous delivery [14]. The final noteworthy mechanical delivery system for DNA that will be discussed is known as a gene gun. This technique involves the physical bombardment of heavy metallic particles coated with plasmid DNA using pressurized gas, similar to the needle-free delivery method [15]. The advantages of gene gun-mediated delivery include significantly reduced plasmid DNA dose requirements for potent immune responses as well as highly efficient DNA delivery to target cells. The reason for this is due to the gene gun’s ability to inject the DNA-loaded particles directly into the cytosol of the target cells, bypassing the cellular membrane. The major drawback to this technology is the cellular damage that can occur from the high velocities imparted on the bombarding particles. Additionally, gene gun systems are expensive, as are the conventional gold particles used to load the plasmid DNA for bombardment. Furthermore, in order to deliver DNA to target organs, surgery is required because gene guns are limited to a narrow and shallow area of effect for delivery [16].

Although mechanical delivery methods for DNA have been shown to be effective, they suffer from the need for expensive equipment, excessive cellular damage, or both. For this reason, non-mechanical delivery methods are highly favored. Since high transfection efficiency is crucial for DNA vaccines to be effective, viral delivery vectors are most prominent. Millennia of natural selection enabled the evolution of efficient viruses capable of subverting the human body’s defense mechanisms, resulting in highly effective gene transporters [17]. These same viruses can be cultivated, manipulated and designed for intentional delivery of a DNA payload. However, due to viral safety concerns including immunogenicity, reversion to virulence, and insertional mutagenesis associated with viral vectors, a prominent research interest is the development of non-viral delivery vectors for DNA vaccines. In order to generate a robust and effective immune response from the DNA vaccine, it also requires specific targeting to lymphoid tissue and organs, and even more specific to dendritic cells (DCs), the most potent type of APC [18]. An additional challenge is the difficulty in achieving high transfection efficiency while inducing DC maturation and antigen presentation. These challenges can be addressed with two main technologies: first, nanocarriers designed to protect the DNA payload and facilitate its transportation to target cells, and second, adjuvants designed to enhance the immune system and boost the body’s immune response to the antigens contained within the vaccine.

Vaccines can be administered a number of ways including oral, intramuscular, intravenous, intradermal, intranasal, topical, and intratumoral. Based on the target tissue and mode of administration, the material composition and chemistry of the DNA nanodelivery system can be designed and tuned to improve therapeutic efficacy for specific environment and applications [19]. Whether it be lymph nodes, the mucosa-associated lymphoid tissue (MALT), or tumor tissue itself, different nanocarriers can be prepared, the specifics of which will be discussed in this review. Regardless of the application, all NP technologies seek to achieve safe, efficient, and controllable DNA vaccine delivery, while maximizing encapsulation efficiency, minimizing toxicity, and increasing immune response potency [20]. This review focuses on recent progress in nanomaterial technology that enhances the efficacy of DNA vaccinations through both improved nanocarrier and adjuvant design.

## 4. Nanotechnologies to Tackle Delivery Challenges of DNA Vaccine

**Figure 2:**
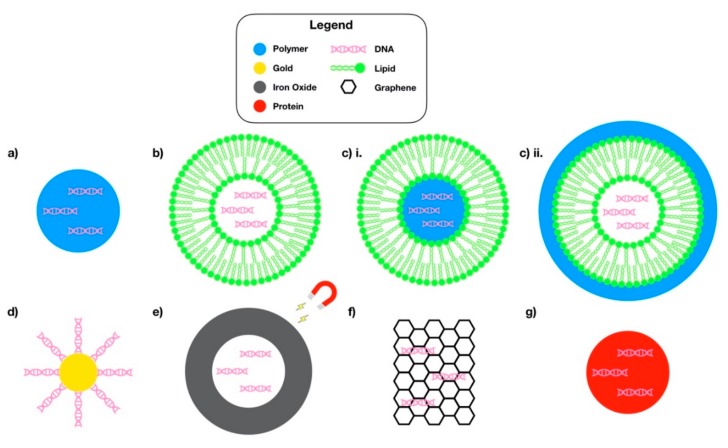
Schematic representations of nanoparticle designs for DNA vaccine delivery. (**a**) Polymer nanoparticle; (**b**) Lipid nanoparticle; (**c**) Lipid-polymer hybrid nanoparticle—(i) DNA-polymer complex core encapsulated in lipid shell, and (ii) Lipid nanoparticle encapsulating DNA coated with polymeric material; (**d**) Gold nanoparticle; (**e**) Superparamagnetic iron oxide nanoparticle; (**f**) Graphene; and (**g**) Protein-DNA complexed nanoparticle/virus-like particle-DNA complex. Not drawn to scale.

Nanoparticles (NPs) implemented as delivery vehicles provide protection of the payload, whether that be vaccine antigens, proteins, drugs, or nucleic acids, against degradation from harsh environmental conditions encountered during transportation to target cells [21]. NPs are in the range 10–500 nm, which enables them to be readily taken up by cells, as well as to avoid RES clearance. NP delivery systems offer the ability to improve the immunogenicity of the DNA payloads, minimize toxicity, enable targeted delivery to APCs, enhance DNA uptake and nuclear entry, and improve overall antigen-specific immune responses [22]. Because of the non-viral nature of nanoparticle delivery systems, they can offer beneficial properties including favorable biocompatibility and biodegradability as well as a more favorable safety profile compared to their viral counterparts if they are appropriately designed. NP delivery systems are also favorable in terms of design and fabrication since they are easier to process and modify in comparison to live or attenuated viral delivery vectors. Moreover, NP technologies offer tunable surface properties through ligand surface modifications to target specific tissue or cells. In particular for vaccine delivery, NP platforms are favorable because they demonstrate an enhanced size-dependent lymphatic uptake associated with particles smaller than 100 nm in diameter, resulting in higher transfection efficiency in APCs within lymph nodes [23,24]. NPs also demonstrate increased uptake across the epidermis and mucosal tissue to reach the MALT [25]. Depending on the materials used in the design of NP-mediated DNA vaccine delivery systems, their behaviors and properties will vary dramatically. Figure 2 outlines various NP systems for DNA vaccine delivery that are described in this review. The following sections will discuss the different materials used for DNA vaccine delivery systems and their suitable applications.

### 4.1. Polymer Nanoparticles.

**Figure 3:**
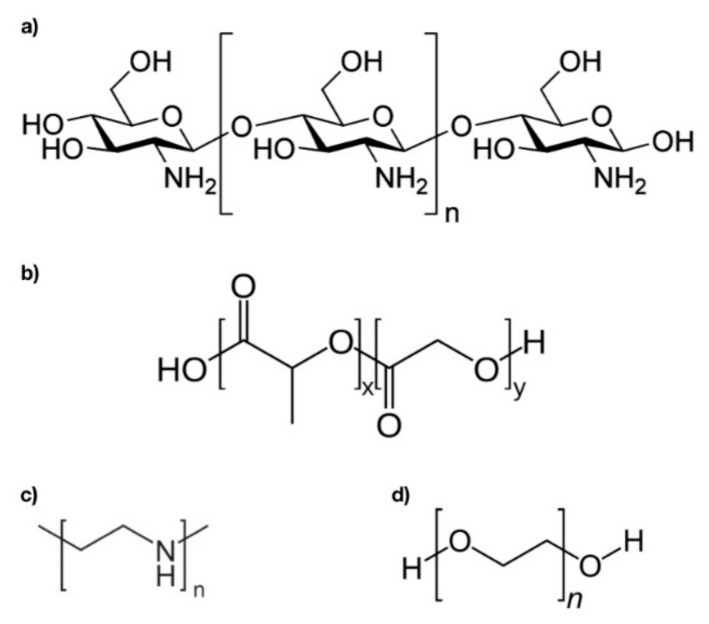
Chemical structures of polymer materials for DNA vaccine delivery. (**a**) Chitosan; (**b**) Poly(lactic-*co*-glycolic acid) (PLGA); (**c**) Polyethylenimine (PEI); (**d**) Poly(ethylene glycol) (PEG).

Polymer NPs are amongst the most widely investigated materials for nucleic acid delivery platforms due to their favorable safety profile, versatility, and ability to enhance immune responses [26,27,28,29,30]. The most common polymeric NPs for DNA vaccine delivery include chitosan, poly(lactic acid) (PLA), poly (glutamic acid) (PGA), and poly(lactic-*co*-glycolic acid) (PLGA) [26,27]. The rate of release of these polymer NPs can be controlled by designing the chemical structure of the particle to behave specifically in different environments. For instance, a pH-stimulated control system would involve having a structure that modulates permeability through dissociation of different surface ligands with changing pH. Based on the chemistry and biological effects inherent to different materials, the functionality and suitable applications for different polymer NPs vary significantly [28]. Figure 3 outlines several chemical structures of common polymers used to develop NP-systems for DNA vaccine delivery.

**Figure 4:**
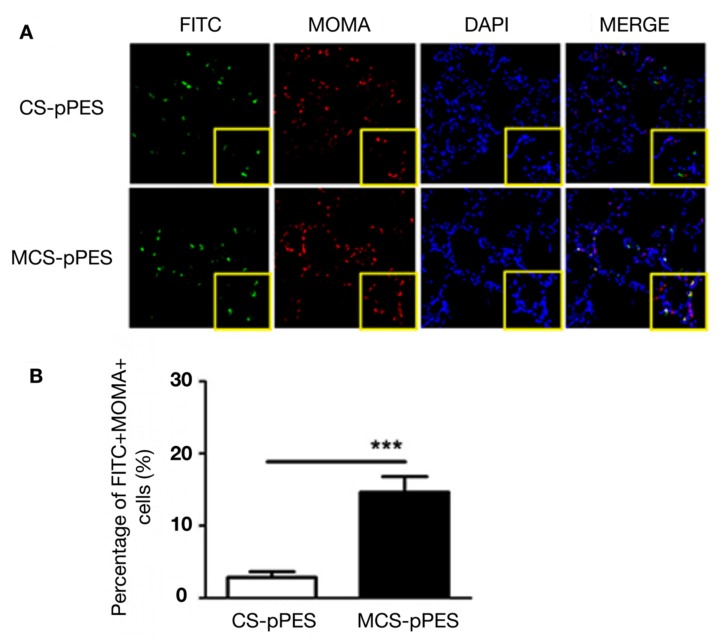
Mannosylation of chitosan nanoparticles (MCS NPs) resulting in enhanced alveolar macrophage targeting for delivery of tuberculosis DNA vaccine compared to regular CS NPs. (**A**) Fluorescent confocal microscopy images of immunized mice lung cross-sections indicating increased uptake of DNA (FITC+, green) within macrophages (MOMA+, red) of MCS NPs compared to CS NPs. (**B**) Quantification of transfection efficiency of DNA NPs (FITC+, green) in alveolar macrophages (MOMA+, red), calculated as a percentage of FITC+MOMA+cells compared to all MOMA+cells. Data expressed as the mean ± SEM from three repeated experiments (*n* = 3). ***p < 0.001. Reprinted from [44], which is an Open Access article distributed under the terms of the Creative Commons Attribution-NonCommercial-NoDerivatives License (http://creativecommons.org/licenses/by-nc-nd/4.0/).

Chitosan is an abundant polysaccharide that is a biocompatible, biodegradable, minimally toxic polymer suitable for biomedical applications [29,30,31]. Specifically for DNA vaccine delivery, chitosan is an appealing material due to its cationic nature, enabling for electrostatic binding to the anionic structure of DNA forming polymer-DNA complexes that provide protection to the DNA against degradation from enzymes. Chitosan is also highly insoluble, inert, and non-immunogenic, with favorable mucoadhesive properties facilitating vaccination strategies via mucosal routes. Chitosan has also been reported to possess natural adjuvant effects, capable of inducing DC maturation through the stimulation of type I interferon (IFN) release which has the added benefit of inducing antigen-specific Th1 responses [32]. Chitosan-based NP delivery technology has been developed over the years and has resulted in numerous developments including a potential therapeutic anti-tumour human papillomavirus (HPV) DNA vaccine [33], treatments for influenza A [34,35], viral myocarditis [36], and a host of animal-borne diseases including Newcastle disease virus (NDV) [37,38,39,40], Nodavirus [41], and Trueperella pyogenes [42]. As mentioned previously, one method to improve the efficacy of DNA vaccines is through targeted delivery to APCs which can be achieved through the functionalization of NPs. The surface of DCs and macrophages express large quantities of mannose receptors and are thus easily targeted by decorating the surface of NPs with mannose structures [43]. In one study, a multi-T epitope DNA vaccine against *Mycobacterium tuberculosis* was developed using mannosylated chitosan NPs as the delivery vector [44]. The results indicated effective targeting of macrophages, which aligned with the potent induction of antigen-specific T-cell responses, as shown in Figure 4. Chitosan has also been shown to have immune-enhancing adjuvant effects when used in conjunction with DNA vaccines [45]. Specifically, it was shown that chitosan promotes DC maturation through induction of type I interferons which consequently enhances antigen-specific T helper 1 (Th-1) responses [32]. In another study, chitosan was used as a delivery vehicle for DNA encoding chicken interleukin-2 (ChIL-2), which possesses the adjuvant potential to induce the activation and proliferation of T cells. This was tested alongside a DNA vaccine for NDV, demonstrating that the co-delivery of ChIL-2 resulted in enhanced protective immunity against NDV [46]. Chitosan nanoparticles were combined with human serum albumin (HAS) capable of enhancing transfection efficiency and improving DNA–chitosan interactions in order to develop a mucosal vaccine against the hepatitis B virus. The nanoparticles were able to induce significant humoral and mucosal responses against hepatitis B virus [47].

Poly(lactic-*co*-glycolic acid) (PLGA) is one of the most widely used polymer materials for drug delivery systems largely due to its FDA approval and enabled by its biodegradability, biocompatibility, and easily tunable physical properties [48]. PLGA NPs release their payload through a hydrolysis process that is slow and is initialized with a burst release in which the payload quickly diffuses out to the surrounding environment. This behavior of drug release for PLGA renders it necessary for chemical modification of the PLGA structures to achieve controlled release of the payload, both spatially and temporally [49]. In the past, PLGA microparticles were commonly studied for use as DNA vaccine delivery vehicles, however they suffer from a number of limiting weaknesses. Upon degradation, PLGA microparticles acidify the microenvironment, destabilizing homeostasis. PLGA microparticles also fail to provide robust protection for their payloads from enzymes, and also attenuate the immunogenicity of delivered vaccines [34]. To address these issues, PLGA NPs were combined with other polymers to make composite systems to increase the stability of the formulation. DNA vaccine-encapsulated PLGA NPs have been developed against diseases including NDV [50], Foot-and-mouth disease virus (FMDV) [51], and *Streptococcus agalactiae* [52]. The composite NP system was developed, composed of PLGA and polyethylenimine (PEI), a cationic polymer widely studied for use as a DNA delivery carrier. This PLGA-PEI NP system was used to deliver a DNA vaccine encoding Rv1733c, a *Mycobacterium tuberculosis* latency antigen, as a primer prior to administration of a Rv1733c protein boost [53]. The results demonstrated that DNA vaccine-encapsulated PLGA-PEI NPs stimulated DC maturation and induced the secretion of IL-12 and TNF-α. In conjunction with the protein boost, the DNA vaccine was shown to enhance T cell proliferation and IFN-γ secretion in vivo, demonstrating strong cell-mediated immunity against the target antigen.

**Figure 5:**
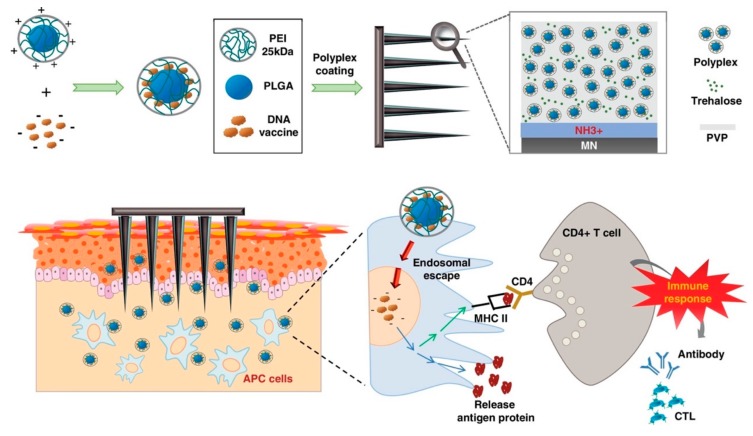
Illustration of PLGA/PEI/pH1NI polyplex coated-stainless steel microneedles for intradermal vaccination. The microneedle system delivers the nanoparticle-DNA complexes across the stratum corneum, into the immune cell-rich region of the epidermis. This facilitates the targeted transfection of antigen-presenting cells that activate cytotoxic T lymphocytes via MHC molecules, stimulating the induction of cell-mediated and humoral immunity to the target disease. Reprinted with permission from H Seok, Journal of Controlled Release, published by Elsevier, 2017 [62].

Polyethylenimine (PEI) is versatile with material properties and behavior that varies greatly with molecular weight and the degree of branching [54]. High molecular weight (MW) PEI, which is generally branched in structure, results in higher transfection efficiency along with higher cytotoxicity. The primary reason for this is because, with higher MW PEI, there is a higher density of amine groups, which results in higher protonation potential. Highly charged polymers are favorable for high transfection efficiency because of enhanced nucleic acid condensation and cellular transfection through the proton sponge effect-mediated endosomal escape mechanism [55]. The toxicity generated from high MW PEI results from PEI NPs aggregating at the surface of cells upon interaction. Conversely, low MW PEI, specifically with a linear structure, possesses a lower surface charge which reduces its cellular toxicity. However, it provides lower transfection efficiency due to its inability to form stable structures with DNA, and protect it from enzyme attack and exposure to harsh biological environments. In order to improve the transfection efficiency of PEI NPs while minimizing toxicity, modification strategies can be applied including conjugation of high MW weight branched PEIs with polysaccharides, hydrophilic polymers, disulfide bridges, and lipid moieties [56]. For the purpose of DNA vaccination specifically, PEI has been used to encapsulate DNA encoding hemagglutinin (HA) from influenza A H5N1 for intranasal immunization which generated high levels of HA-specific IgG A antibodies and protective humoral and cell-mediated immunity against H5N1 challenge in mouse models [57]. Recently, β-cyclodextrin-PEI600, a cationic polymer, was used to develop a modified bacterial delivery system for oral DNA vaccination as an in vivo platform for cancer immunotherapy [58]. By coating live attenuated bacteria with cationic polymer-DNA NP complexes, the bacteria were able to more effectively escape phagosomes and were also provided significant acid tolerance, which was useful in the stomach and intestines. This resulted in a greater dissemination of bacteria into circulation after oral administration. Most importantly, remarkable T cell activation and cytokine production were achieved, as well as successful inhibition of tumor growth through oral delivery of DNA vaccines encoding autologous vascular endothelial growth factor receptor 2 (VEGFR2). PEI has also been used in conjunction with microneedle technology which has demonstrated the ability to generate more potent immune responses than conventional delivery methods. A DC targeting transcutaneous DNA vaccine delivery system was recently developed for malignant melanoma therapy in which mannosylated grafted cell-penetrating peptides were conjugated to low molecular weight PEI (termed as CPP-PEI_1800_-Man), and complexed with TRP-2 pDNA to form nanocomplexes for microneedle-assisted transcutaneous immunization [59,60]. The mannose modification improved DC-targeting delivery and the CPP enhanced the transfection efficiency of pTRP-2 in DCs. With the aid of microneedles, the polyplexes of CPP-PEI_1800_-Man/pTRP-2 promoted TRP-2-specific cellular immune responses, resulting in potent tumor growth inhibition and prolonged survival time of B16-xenografted mice. In this study, the solid microneedles served as a penetration enhancer by breaching the stratum corneum, a layer of the skin that functions as a physical barrier against the external environment which has proven to be a significant barrier for intradermal vaccination. In another recent study, an intradermal pH1N1 DNA vaccine delivery system was developed using microneedles coated with polyplexes containing PLGA/PEI NPs as shown in Figure 5. Immunization results showed that coated polyplexes on microneedles induced greater humoral immune responses than that of intramuscular polyplex delivery and naked pH1N1 DNA plasmid delivered by a dry-coated microneedle [61].

Poly(ethylene glycol) (PEG) is an FDA approved polymer commonly used to surface functionalize NPs [63]. The primary function of PEG is to shield the surface charge of the NPs and provide steric stabilization. These effects reduce charge-associated cytotoxicity, prevent nonspecific interactions with serum proteins as well as renders NPs undetectable to phagocytes, providing protection against RES clearance. PEG functionalization results in greater systemic circulation time, improved stability in the bloodstream and lower immunogenicity, however, a proven drawback is lower transfection efficiency [64]. In a recent study, a NP composed of the pyruvate dehydrogenase-derived protein, E2, was surface-functionalized with PEG, CpG oligonucleotides, and Toll-like receptor (TLR) 9 agonists that target antigen-presenting cells. The results of the study demonstrated that the PEG coating improved APC uptake and lymph node accumulation of E2 proteins, which could be further improved through conjugation with CpG DNA [65]. Another instance of vaccine enhancement through PEG functionalization involved the use of PEG-coated lipopeptide-DNA complexes. The study demonstrated that a PEG coating improved the biodistribution, exogenous protein expression and immune responses generated from the DNA vaccine. Intramuscular administration of the DNA vaccine resulted in increased levels of ovalbumin (OVA)-specific antibodies and epitope-specific T cell activity in vivo, demonstrating the effectiveness of the system for DNA vaccine delivery as well as the usefulness of PEGylation [66].

The studies detailed above demonstrated that polymeric nanomaterials offer a wide array of functionality attributed to their versatility in regard to their composition and molecular weight. Polymers offer good biocompatibility and safety profiles, positioning these materials as great candidates for the design of non-viral delivery vectors for DNA vaccines. Despite the generally safe nature of polymer-based delivery systems, the limited transfection efficiency they provide restricts their wide spread adoption and progression in clinical trials and beyond. As polymer science research continues to advance, non-viral and polymer-based DNA vaccine systems will continue to improve and eventually will provide comparable transfection performance to viral vectors that are the current standard in vaccine technology.

### 4.2. Lipid Nanoparticles

For DNA vaccines as well as general nucleic acid delivery applications, lipid-based NPs are another prominent class of material used as non-viral vectors [67]. Liposomes have been a widely studied lipid-based delivery system for nucleic acid delivery due to their high transfection and encapsulation efficiency, as well as their easily tunable surface properties [68]. However, there are several challenges that hinder the transition of liposomes to clinical studies which include toxicity, nonspecific immunogenicity, instability in circulation, and rapid clearance from the body. Liposomes are cationic nanoparticles consisting of phospholipids and cholesterol capable of binding and encapsulating DNA. The high transfection efficiency of liposomes and lipid-based delivery systems is generally attributed to their material compatibility with the lipid bilayers that make up the cell membrane, facilitating cellular entry [69].

Pairing liposomes’ inherent properties with specific surface ligands can create potent targeted delivery platforms. Liposome NPs can be functionalized with shikimic acid, a molecule similar to mannose, capable of binding to mannose receptors and enabling targeted delivery to DCs [70]. In one instance, shikimic acid-functionalized liposome NPs were used to deliver DNA encoding for a melanoma-antigen. Treatment with this vaccine stimulated prolonged immune responses against the target, which was attributed to effective DC targeting that has been notoriously difficult to do in vivo. Prophylactic immunization in mice resulted in long-lasting and complete protection against a lethal melanoma challenge, enabling mice to live tumor-free for 100 days. When used in a therapeutic capacity, the DNA vaccine was able to inhibit tumor progression significantly, demonstrating the system’s ability to generate both potent primary immune responses along with long-lasting memory responses [71].

Lipid-DNA NP complexes have also been used as vaccine adjuvants. Lipid NPs complexed with non-coding plasmid DNA were implemented as an adjuvant for a whole-inactivated influenza A virus (IAV) vaccine in rhesus macaques. The results demonstrated the ability of the lipoplex adjuvant to stimulate increased levels of general IAV nucleoprotein and matrix-1 protein-specific antibodies in addition to greater activation of natural killer (NK) cells. In conjunction with the whole-inactivated IAV vaccine, the lipoplex adjuvant was shown to suppress uncontrolled viral replication and reduce levels of viral RNA in vivo [72].

**Figure 6:**
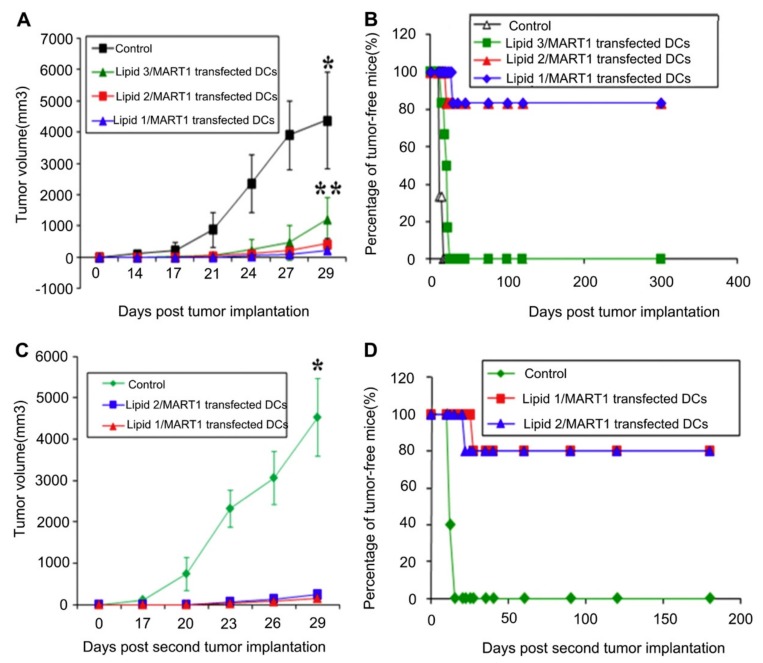
Long term tumor protection induced by immunization with DCs transfected ex vivo with melanoma DNA vaccine delivered via liposome NPs. **A**) Mice were immunized twice with murine-bone-marrow-derived DCs (mbmDCs) pre-treated with DNA encoding a melanoma-associated antigen, p-CMV-MART1, demonstrating the induction of protective immunity against a lethal melanoma tumor challenge. **B**) Percentage of tumor-free mice from the study mentioned above. **C**) The immunized mice that survived the first tumor challenge described above for 120 days were challenged a second time with another lethal melanoma tumor. A notable memory response to the melanoma DNA vaccine was observed through prolonged tumor growth inhibition after the second lethal challenge. **D**) Percentage of tumor-free mice remaining post-second lethal melanoma challenge. Data expressed as the means +/- SD for *n* = 6 tumors (*P < 0.005 vs. tumor sizes for lipoplexes of lipids 1–3, **P < 0.005 vs. tumor sizes for lipoplexes of lipids 1 and 2). Reprinted with permission from R Srinivas, Biomaterials, published by Elsevier, 2012 [78].

Various other lipid-based particles have been investigated for DNA vaccine delivery applications in order to overcome the weaknesses associated with liposome-based systems. One such material is the niosome, consisting of cholesterol or cholesterol-like molecules and non-ionic surfactants which form a highly stable bilayer vesicle through protection against lipid oxidation [73,74]. The stability of niosomes was shown to be further enhanced through mannosylated surface functionalization which simultaneously allowed for targeted delivery to APCs [75]. Niosomes have demonstrated potential as DNA vaccine carriers for topical epidermal administration against hepatitis B [76] and has also been used in conjunction with hollow microneedles for epidermal vaccination, inducing humoral and cellular immune responses against the antigen encoded in the DNA payload [77]. Another group demonstrated significant induction of long term protective immunity against melanoma through an ex vivo transfection of autologous DCs with melanoma encoding DNA delivered via liposomes as shown in Figure 6 [78]. From this figure, lipids 1 and 2 are cationic amphiphiles, lipid 1 with mannose mimicking quinic acid head groups, and lipid 2 with shikimic acid head-groups while lipid 3 being their mannosyl analogue. Although ex vivo DC transfection-based DNA vaccines have been proven to be a remarkable discovery at generating potent immune responses for cancer immunotherapy, its suboptimal efficacy and expensive technology open the door for further improvements in this field [79].

Recently in 2018, a lipid NP system for small interfering ribonucleic acid (siRNA) delivery to treat Transthyretin amyloidosis, a peripheral nerve disease, was approved by the FDA [80]. This was the very first siRNA treatment to be approved in history, which was a great step forward in the development of gene therapy technology. Although this system utilized siRNA, not DNA, the two do share many commonalities. As such, the approval of this treatment is still a testament to the efficacy and potential of lipid NP systems for nucleic acid delivery. There is ongoing research seeking to adapt lipid NP technology developed for siRNA delivery to larger payloads, like plasmid DNA, which renders it likely that a DNA-based gene therapy delivered with lipid NP is not too far behind [81].

### 4.3. Hybrid Lipid-Polymer Nanoparticles

In the previous sections, the strengths and weaknesses of both polymer-based delivery systems and lipid-based delivery systems were discussed. Polymeric materials offer highly biocompatible, biodegradable, and non-immunogenic properties with the capacity to be easily modified for functionalization to increase stability, and provide controlled release, and targeted delivery. However, polymeric gene delivery vectors generally suffer from poor transfection efficiency and the inability to produce significant exogenous protein expression. Conversely, lipid-based delivery systems offer high transfection efficiency and biocompatibility, however, they are hindered by their large size, toxicity, structural instability, and rapid systemic clearance. Combining these two technologies unlocks the ability to harness the advantages of both polymeric and lipid materials for nucleic acid vaccine delivery while compensating for the opposing material’s weaknesses. Such a delivery system is called a lipopolyplex which consists of a polymer-nucleic acid complexed core encapsulated by a liposome shell. Often the liposome shell is decorated with an outer lipid-PEG layer which functions as a steric stabilizer, preventing immune detection and enhancing the systemic circulation time. The lipopolyplex design provides the synergistic benefits of both polymer and liposomal materials, offering higher transfection efficiency and lower cytotoxicity than other platforms implementing only one or the other [82,83].

In an effort to develop an efficient DNA vaccine delivery system targeting the Peyer patch, plain liposomes were compared to polymer-liposome hybrid NPs. It was demonstrated that oral administration of the chitosan-coated lipopolyplex design was able to generate relevant levels of exogenous green fluorescence protein (GFP) expression throughout the intestine, while a plain chitosan-coated liposome was only able to generate exogenous GFP expression within the upper duodenum. The results showed that the lipopolyplex design induced a prolonged systemic circulation time through decreased enzyme degradation and was, in general, more effective in delivering DNA to target tissue than the non-hybrid NP counterpart [84]. This proof-of-concept study demonstrated the effectiveness of chitosan-coated lipopolyplexes as an oral gene delivery vector for the future developments of DNA vaccines. Despite the advantages of lipopolyplex designs, there has been limited research into the development of lipid-polymer hybrid nanoparticle delivery systems specifically for DNA vaccines. There has been, however, a high volume of research done on lipopolyplexes for mRNA vaccine delivery, likely because of its more favorable safety profile, and the fact that it does not require nuclear entry or genomic integration, rendering the therapeutically relevant generation of exogenous proteins more easily achievable [85,86,87,88].

Lipid-polymer hybrid NPs offer the ability to combine the advantages of both polymer and lipid materials in a single design. Although there is comprehensive research in the development of new materials for efficient DNA delivery, there is a lack of informative studies comparing existing delivery technologies. As such, it is difficult to understand whether polymer NPs or lipid NPs are more effective for DNA vaccination, let alone hybrid systems comprising of the two. To further improve the hybrid NP systems for DNA delivery, comparative studies of different delivery systems should be conducted, and this would provide further understanding to investigate what combination of materials, in terms of composition and proportion, are optimal for the delivery of DNA vaccine.

### 4.4. Inorganic Nanoparticles

Inorganic nanomaterials have attracted significant interest in DNA vaccine delivery applications due to their ease of functionalization, biocompatibility, and well-defined chemistry. Moreover, their thermal and chemical stability facilitates the process of sterilization, which is not possible with other classes of materials [89]. Inherently, inorganic materials have low toxicity and can be easily synthesized with tunable sizes, shapes and aspect ratios. Furthermore, the use of inorganic materials opens up a wide range of targeting and imaging techniques when using magnetic NPs or quantum dots.

**Figure 7:**
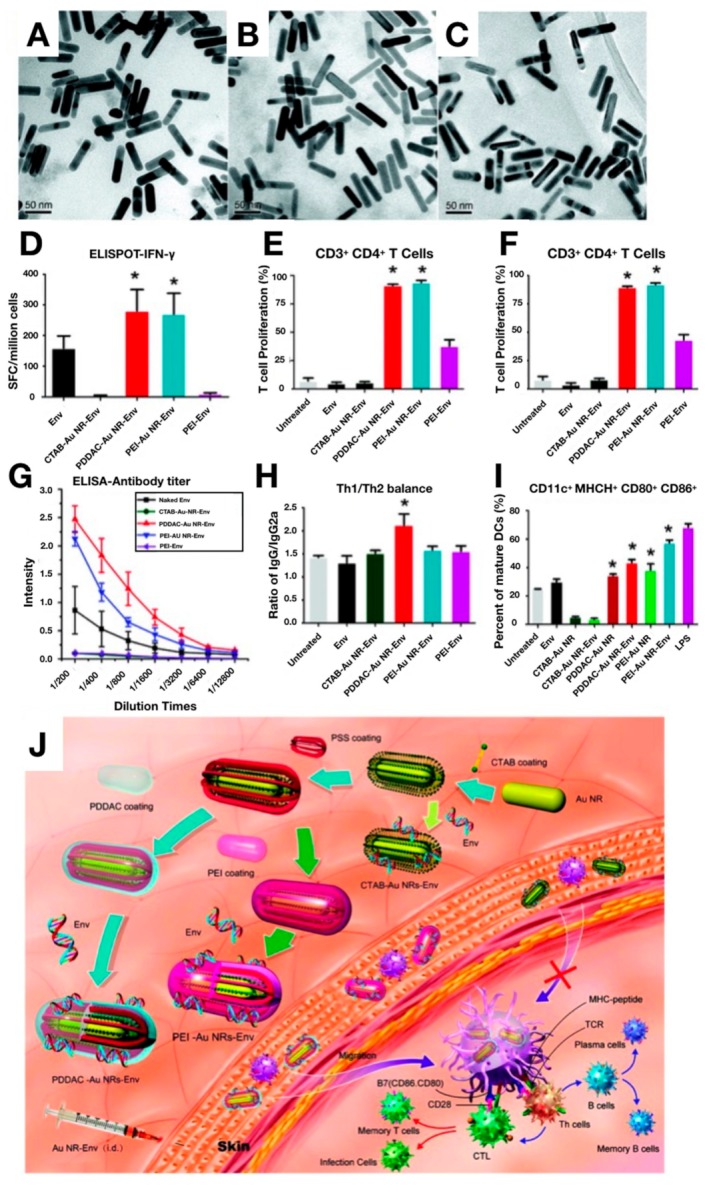
Gold nanorods for use as DNA vaccine adjuvants against HIV. Surface modified gold (Au) nanorods (NRs) imaged with TEM: CTAB-Au NRs (**A**), PDDAC-Au NRs (**B**), and PEI-Au NRs (**C**). (**D**–**I**) Graphs quantifying the immune responses stimulated by Au NRs (**D**) IFN-γ analyzed by ELISPOT. (**E**) CD3^+^CD4^+^ T cell proliferation. (**F**) CD3^+^CD8^+^ T cells proliferation. (**G**) The structural envelope protein (Env) specific antibody titer measurement. The Au NR-based DNA vaccine stimulated humoral dominated immunity. (**H**) Comparing balance of cell-mediated immune response versus humoral immune response stimulated in mice after immunization with Au NR-Env plasmid DNA complexes. (**I**) The effect of Au NRs and the Au NR-Env complex on DC maturation. (**J**) Hypothesized mechanism of action for Au NR vaccine adjuvants. Surface coated Au NRs mixes with Env to form Au NR-Env complexes. Intradermal administration of PDDAC- and PEI-Au NR-ENV complexes into the immune-cell rich region of the skin facilitates transfection of APCs, which process the antigens and migrate to secondary lymph nodes for antigen presentation via the MHC complex to naïve T cells. Cytotoxic T lymphocytes (CTLs) proliferate rapidly and eliminate intracellular pathogens, regulated by activated T helper cell 1 (Th1). It is possible that Th1 polarization occurs first, followed by transformation to Th2 polarization. Activated Th2 cells induce the transformation of B cells into plasma cells which secrete immunoglobulin (IgG). Following this, a fraction of T cells and B cells becomes memory cells that protect the organism against future infection with the same pathogen. It is thought that CTAB-Au NRs inhibits DC maturation, which hinders the immunogenicity of the HIV-1 DNA vaccine. Reprinted with permission from L Xu, Nano Letters, published by American Chemical Society, 2012 [95].

Among the inorganic nanomaterials, gold (Au) NPs are a widely-used material due to their well-defined and versatile surface chemistries, ease of fabrication, and good biocompatibility. They play an important role in the field of vaccination as both a delivery platform, and an adjuvant, capable of reducing toxicity, enhancing immunogenic activity, and providing stability in regard to vaccine storage. A considerable number of papers have been published in the past two decades that are devoted to the use of gold NPs for generating DNA vaccines [90,91,92,93,94,95,96,97,98,99,100]. A recent study demonstrated that gold NPs could be covalently functionalized with a thiol-based structure containing shikimoyl, a DC targeting ligand, and guanidinyl, a ligand that increases transfection efficiency. This functionalized gold NP demonstrated the ability to deliver DNA vaccines to DCs in vivo with high efficiency, inducing prolonged protective immunity against murine melanoma [97]. Using a similar gold NP design, an ex vivo approach to DNA vaccination against murine melanoma was also developed. Gold NPs were conjugated with shikimoyl ligands using a thiol spacer and complexed with DNA encoding a melanoma antigen for ex vivo transfection of DCs. Immunization with the DNA vaccine-transfected DCs induced potent humoral and cell-mediated immune responses, successfully protecting inoculated mice against a lethal dose of melanoma [98]. In a less conventional approach, a DNA vaccine delivery system was developed in which gold NPs were electrically stimulated in order to drive vibrational and dipole-like oscillations capable of increasing the permeability of cell walls, enhancing the transfection of a DNA vaccine. In this study, hepatitis C virus was used as the model disease, demonstrating the induction of significant humoral and cell-mediated immune responses against the virus within immunized mice [99]. Gold NPs have also been used in the development of novel adjuvants [95]. High aspect ratio gold nanorods were functionalized with cetyltrimethylammonium bromide (CTAB), poly(diallyl dimethylammonium chloride) (PDDAC), and PEI for use as a DNA vaccine adjuvant for HIV treatment using HIV-1 Env plasmid DNA as the antigen, as shown in Figure 7. In vivo results demonstrated that the PDDAC- and PEI-modified gold nanorods markedly enhanced humoral and cell-mediated immunity induced against HIV when compared to CTAB-modified gold nanorods and naked plasmid DNA [95]. Specifically, Th1/Th2 ratios were measured to determine the bias of the immune response stimulated by the DNA vaccine. Th1 is the main driver for cellular immunity and in mice stimulates the production of IgG2a, while Th2 is the main driver for humoral immunity and stimulates the production of IgG1. In Figure 7h, it can be seen that the IgG/IgG2a ratio is the largest for the PDDAC-AU NR-Env treatment, especially compared to the Env control group, indicating a Th2-biased immune response. In order to probe the mechanism of action for the vaccine adjuvant effects of the gold nanorods, costimulatory molecules responsible for triggering clonal expansion and differentiation of naïve T cells were measured. Figure 7i indicates that the percentage of mature DCs, through the measurement of CD11c+ MHCII+ CD86+ CD80+ markers, was increased significantly for groups treated with PDDAC and PEI functionalized gold nanorods, but not for CTAB functionalized gold nanorods. This result indicates that the surface chemistry of the gold nanorods heavily influences the resultling adjuvant activity.

**Figure 8:**
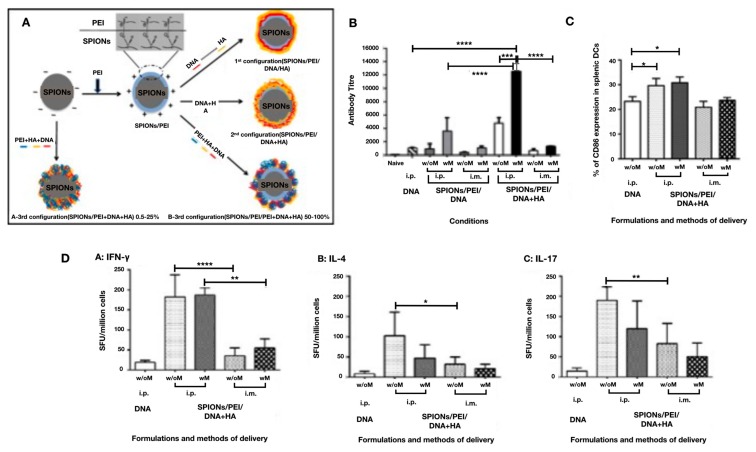
SPION based DNA vaccine delivery system enhanced using an external magnetic field. (**A**) Illustration of superparamagnetic iron oxide nanoparticles (SPIONs) functionalization with polyethylenimine (PEI), and hyaluronic acid (HA) in different synthesis sequences for use as carriers for malaria DNA vaccine encoding *Plasmodium yoelii* merozoite surface protein MSP1-19 (VR1020-PyMSP1-19). Reprinted with permission [110]. (**B**) Results of Elisa assay demonstrating the induction of antibody responses by SPIONs/PEI/DNA + HA, SPIONs/PEI/DNA, or naked DNA, through different routes of administration, either intraperitoneal (i.p.) or intramuscular (i.m.). (**C**) Activation of dendritic cells in the spleen post injection with SPIONs/PEI/DNA + HA complexes via i.p. or i.m. administration (naked DNA via i.p. only), with or without the application of a magnetic field. (**D**) Results of ELISpot assays demonstrating antigen-specific T cell responses induced by the SPIONs/PEI/DNA + HA complexes in vivo. Data expressed as antibody titer mean ± SD of 2 individual experiments. Statistical significance was designated as * *p* ≤ 0.05, ** *p* < 0.01, *** *p* < 0.001, **** *p* < 0.0001, ((w/M) with magnet, (wo/M) without magnet). Reprinted from [106], which is an Open Access article distributed under the terms of the Creative Commons Attribution-NonCommercial-NoDerivatives License (http://creativecommons.org/licenses/by-nc-nd/4.0/).

Ferric NPs are another class of materials that has been widely implemented as gene delivery vectors due to their favorable properties including low toxicity, inexpensive cost, ease of surface functionalization, and the ability to bind biological materials. Ferric materials can also provide unique magnetic properties that have been exploited for applications in imaging [101], tumor ablation [102], and active targeting for drug delivery through the control of external magnetic fields [103]. Superparamagnetic iron oxide NPs (SPIONs) specifically have been broadly used for drug delivery applications and have been widely successful [104]. SPIONs’ magnetic properties allow for use of external magnetic fields to guide the NPs and bounded drug to target regions and tissue within the body for precise controlled accumulation of the therapeutics [105]. A SPION-based DNA vaccine delivery system was functionalized with PEI and hyaluronic acid (HA) to improve the stability and APC targeting ability of the vector, as shown in Figure 8 [106]. This delivery system was used in conjunction with an external magnetic field focused around the injection site in order to stabilize local SPION concentrations to achieve controlled and sustained exposure to the vaccine in the target area. In vivo immunization with the SPION-PEI-HA malaria DNA vaccine induced noticeable humoral and cell-mediated immunity, showing that the application of the external magnetic field increased antibody production, however, had no additional effect for the T-cell response [107]. One recent study showed the use of multicomponent NPs, combining the safe and biocompatible properties of poly(ß-amino ester) (PBAE) and the magnetic properties of SPIONs. Different configurations of these NPs were synthesized and tested in vitro to evaluate the transfection efficiency of GFP DNA using these designs. Remarkably, the multicomponent NPs provided higher transfection efficiency than SPIONs alone, and could be further enhanced with the application of a magnetic field. Through this study, the PBAE-SPION platform demonstrated the potential to be an effective delivery system for DNA vaccines upon further development [108]. SPIONs offer the unique property of manipulating the behavior, functionality, and delivery of DNA vaccines through the use of an externally applied magnetic field. Although the technology has seen widespread interest and success in the fields of imaging and tumor ablation, the application to drug delivery is limited. Specifically, for DNA vaccination, there has not been much research conducted on SPION technology, meaning the full potential of SPIONs for this application has not yet been realized, which opens up possibilities for further investigation in this domain [109].

Other types of nanomaterials such as silver (Ag) NPs, layered double hydroxide (LDH) NPs, and calcium phosphate NPs have also been used as DNA vaccine delivery vectors and adjuvants [109,111,112,113]. Recently, silver NPs and LDH NPs, commonly known as hydrotalcite-like materials and anionic clays, have received considerable attention as vaccine delivery systems due to their minimal cytotoxicity and ability to provide robust protection of loaded plasmid DNA [111,114,115]. A recent study demonstrated the use of hollow Ag-SiO_2_ NPs as a delivery vehicle for a Newcastle disease virus (NDV) DNA vaccine, encoding F gene plasmid DNA (pFDNA). Intranasal immunization of pFDNA-Ag-SiO_2_ NPs was capable of sustained release of the vaccine, inducing strong humoral and cellular immunity [115]. In other recent studies, silica-Mg/Al-LDHs core-shell NPs have been investigated as immunoadjuvants [114]. When co-cultured with macrophages, the silica-Mg/Al-LDHs NPs promoted IFN-γ and IL-6 cytokine production and enhanced CD86 and MHC II expression in a dose-dependent manner. Furthermore, in vivo immunization of mice indicated that a hepatitis B virus DNA vaccine loaded into SiO_2_-LDH NPs not only induced much higher serum antibody responses than naked plasmid DNA, but also promoted T-cell proliferation and skewed T helper cells towards Th1 polarization.

### 4.5. Virus-Like Particles

Virus-like particles (VLPs) have been developed to circumvent the problems associated with conventional virus-based vaccination strategies including immunogenicity, reversion to virulence and insertional mutagenesis [116]. VLPs are particles that closely resemble viral structures but are inherently safer because they do not contain viral genetic material [117,118]. Appropriately designed VLPs can bind to mucosal surfaces and resist degradation from digestive enzymes as well as highly acidic and alkaline pH conditions within the digestive tract. VLPs possess excellent adjuvant properties and induce an innate and cognate immune response. They are also a safer alternative to attenuated viruses as they do not replicate and are not infectious [119]. VLPs offer advantages of morphological uniformity, biocompatibility, and ease of functionalization [111]. To incorporate nucleic acids into VLPs, two strategies have been developed [120]. In the first approach, osmotic shock is induced by submerging the VLPs in a low ionic strength buffer. This increases the space between surface subunits and allows the nucleic acid to enter through an electrostatic pull from the VLPs’ internal positive charge. The second approach involves the self-assembly of the VLP subunits in the presence of nucleic acids where the encapsulation occurs through electrostatic interactions and phase separation mechanisms. Through these methods, double-stranded DNA up to 4 kb can be encapsulated within VLPs [120]. Most recently, a complex self-assembly reaction buffer system was capable of encapsulating up to 17 kb of supercoiled plasmid DNA within a VLP [121].

**Figure 9:**
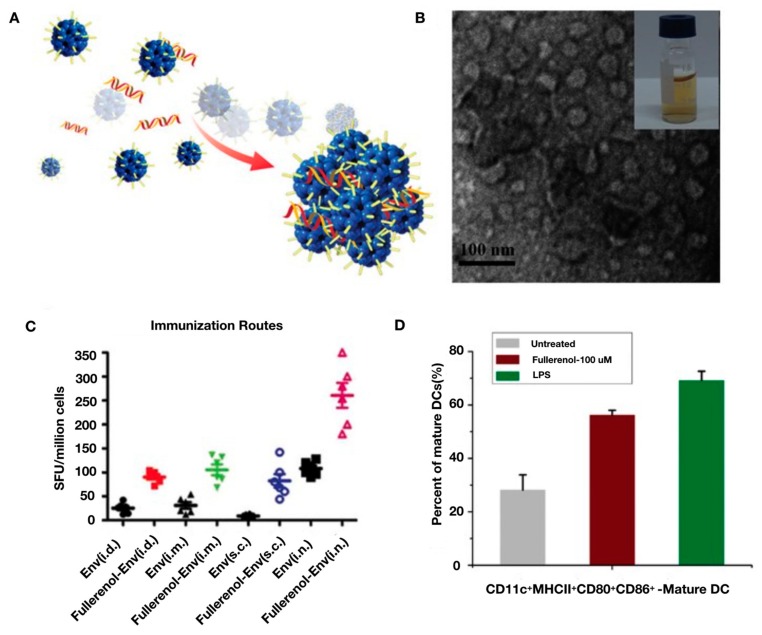
Fullerenol-based virus-like nanoparticles for DNA vaccine delivery. (**A**) Illustration of HIV-1-associated envelope protein, gp145 (Env), plasmid DNA encapsulated during the self-assembly of fullerenol into a virus-like particle. The blue speheres represent fullerenol molecules, the red and yellow wavy structures represent DNA strands, and the short linear sitcks represent hydroxyl groups. (**B**) Fullerenol-Env complex imaged with TEM. (**C**) IFN-γ production induced by immunization with different fullerenol-Env configurations delivered through various immunization routes, including intradermal (i.d.), intramuscular (i.m.), subcutaneous (s.c.) and intranasal (i.n.) injections or inoculations. (**D**) DC maturation induced by immunization with fullerenol. Reprinted with permission from L Xu, Advanced Materials, published by John Wiley and Sons, 2013 [122].

Fullerenols, or polyhydroxy fullerenes, are capable of self-assembling into virus-like particles with an average size of 40 nm, designed with dual functionality. First, as a nano-adjuvant capable of enhancing immune responses to a targeted vaccine, and second, as a high-loading capacity DNA delivery vector. Fullerenols have been studied as a proof-of-concept DNA vaccine delivery system, using HIV-1 DNA antigen as a model antigen for immunization in mice [122]. In vitro, these VLPs significantly enhanced DNA transfection of enhanced green fluorescent protein (EGFP) DNA plasmid in human embryonic kidney 293 (HEK293) cells. In vivo, the VLP NPs enhanced both innate and cellular immunity through various immunization routes as shown in Figure 9. These fullerenol-based VLPs also induced DC maturation and triggered polyvalent immunities via the activation of multiple Toll-like receptor (TLR) signaling pathways. These findings indicated that fullerenols designed as virus-like particles can be used as effective vaccine nanoadjuvants.

VLPs from different origins differ in their stability, therefore the shelf life of different VLPs needs to be studied to get a comprehensive understanding of the materials used [111]. The NPs’ physical properties such as size, shape, composition and surface chemistry can greatly influence toxicity as well as clearance time from the body. Thus, a detailed evaluation of toxicity and complete in vivo biodistribution is necessary before implementing VLPs as a DNA vaccine-based nanomedicine [111].

### 4.6. Protein-Based Nanoparticles

Peptide-based nanocarriers are a favorable platform for DNA vaccine delivery due to their inherent biocompatibility, biodegradability, and generally low cytotoxicity [123,124]. Although proteins can effectively bind small molecule drugs, they have a particularly low loading capacity for DNA, and also lack the cellular specificity which limits the platform’s overall effectiveness for vaccination. For this reason, there has been far less research conducted for protein-based DNA delivery systems compared to polymers, lipids, and or other materials mentioned previously in this review.

For gene therapy applications in general, an extremely well-studied protein for nucleic acid delivery is protamine. Protamine is a cationic protein whose structure efficiently complexes plasmid DNA and possesses several nuclear localization signaling regions that mediate transport across the nuclear membrane. Thus unsurprisingly, protamine is a popular delivery platform for DNA vaccines. An additional appeal of protamine as a DNA delivery vector is that protamine sulfate has been approved by the FDA to reverse the effects of heparin, thereby being a safe and convenient material for the development of new vaccines. In one study, mannosylated protamine sulfate (MPS) was complexed with plasmid DNA encoding gastrin-releasing peptides (GRP). GRP is an autocrine growth factor that can stimulate tumor progression in certain cancers when interacting with GRP receptors and are thus a potential target for cancer immunotherapy. The results of the study demonstrated that the mannose functionalization of the protamine particles resulted in higher transfection efficiency in APCs compared to the non-mannose-functionalized counterparts. In vivo studies demonstrated that the MPS-GRP system induced significant levels of anti-GRP antibodies, preventing GRP binding to GRP receptors and resulting in effective tumor growth inhibition [125]. In another study, a DNA delivery vector was developed by functionalizing protamine with gelatin B, a protein derived from collagen. For this study, model DNA from salmon testes were used. Gelatin B functionalization serves to facilitate endosomal escape through a pH-dependent pathway. Specifically, gelatin B has an isoelectric point between 4.8 and 5.2, which renders it negatively charged in physiological pH, allowing it to capably bind to the cationic structure of protamine [126]. When gelatin B enters an endosome, the pH falls below the isoelectric point, causing the protein to be positively charged and resulting in endosomal escape through the proton sponge effect and consequential release of the payload into the cytosol of the cell. Thus, when gelatin B is complexed with protamine for DNA delivery, both endosomal escape and nuclear entry are enhanced, enabling high transfection efficiency of the DNA payload. There has yet to be in vivo results to validate this delivery platform. However, in vitro results have demonstrated that gelatin-B-protamine-DNA complexes display low cytotoxicity and appear to be safe from a cellular standpoint [127].

**Figure 10:**
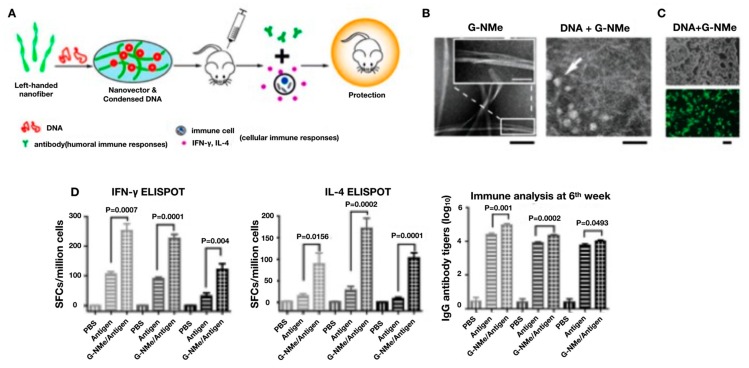
Peptide-based nanofibrous hydrogel HIV DNA vaccine. (**A**) Schematic of synthesis, immunization and immune response generated from peptide-based nanofibrous hydrogel HIV DNA vaccine. (**B**) G-NMe nanovectors imaged with TEM. Scale bar: 100 nm (black); 50 nm (white). (**C**) Fluorescence images of 293 T cells transfected by G-NMe nanovector/EGFP plasmid. Scale bar: 100 μm. (**D**) Results of ELISPOT assays indicating cellular immune responses induced by GNMe/DNA complexes. Data expressed as the mean ± standard deviation of titers (log 10) or spots. Reprinted with permission from Y Tian, Nano Letters, published by American Chemical Society, 2014 [128].

Recently, a peptide-based DNA vaccine nanovector was developed which induced enhanced cellular and humoral immune responses against HIV via intramuscular, intradermal, and subcutaneous injection [128]. Four amino acid backbone (glycine–phenylalanine–phenylalanine–tyrosine) peptide, abbreviated as G-NMe, modified with naphthalene acetic acid (N-terminal) and an N-methyl group (C-terminal) was shown to rapidly assemble into nanofibers in the presence of alkaline phosphatase. The enhanced immune stimulation generated by the G-NMe nanovectors is hypothesized to be a result of the left-handed structure of the nanofibers which enables more efficient condensing of DNA, providing more potent protection from degradation, improving the overall transfection efficiency of the DNA vaccine, as shown in Figure 10.

Protein-based delivery systems have seen limited use for DNA delivery specifically due to their low loading capacity, which restricts their overall therapeutic efficacy. For the same reason, greater success with siRNA delivery has been achieved because they are much smaller in size, enabling higher concentrations of loading, and enabling greater therapeutic effect. Despite these challenges, further research should be continued to improve the loading capacity of DNA in proteins, because they offer the unique ability to serve a dual purpose of binding and delivering DNA, but also providing additional functionality, either acting as an antigen or adjuvant itself, or mediating transport across cell membranes or nuclear membranes.

## 5. Clinical Trials on DNA Vaccine Technology

Based on existing literature, there have been only a few nanomaterial-based DNA vaccine delivery systems that have successfully progressed to clinical trials, and none have been approved for use thus far [6]. Results of clinical trials in humans have demonstrated that current DNA vaccine technologies are capable of inducing a small degree of specific humoral and cell-mediated immune responses, however, they are not potent enough for therapeutic relevance. One possible reason for the discrepancy between the therapeutic success in animals and humans is the challenging need to significantly increase the quantity of DNA vaccine-based responses by several orders of magnitude to achieve similar immunogenic responses to those seen within smaller animal models [7].

One method to address this challenge is through the use of adjuvants. Similar to inactivated and subunit protein-based vaccines, DNA vaccines can also benefit from the use of adjuvants to enhance the protective immune responses generated. One nanomaterial-based adjuvant that was studied in a clinical setting is Vaxfectin, a cationic liposome that can ionically bind to DNA and potentiate the immune response against H5N1 influenza-associated proteins including HA, nucleoproteins, and viroporins [7,123]. This works through upregulating immune cell recruitment, increasing antigen presentation and altering the immune microenvironment through modulation of cytokine secretion patterns [123]. Notably, Vaxfectin does not increase transfection efficiency of the DNA payload, and thus is considered an adjuvant and not a delivery system. Through a Phase I clinical trial, Vaxfectin-adjuvanted DNA vaccination demonstrated the ability to induce immune responses against influenza A virus H5N1 to similar levels generated by inactivated protein-based vaccines. Furthermore, the vaccine was well tolerated, demonstrating acceptable safety standards suggesting that Vaxfectin-adjuvanted DNA vaccination is potentially viable for rapid implementation for pandemic control [124]. Vaxfectin was also used in a recent study that reported the results of Phase I clinical trial for a tetravalent dengue DNA vaccine. The study demonstrated that the DNA vaccine was both safe, and capable of inducing protective cell-mediated immunity against dengue virus [129].

Another major application of DNA vaccine technology is for the use of cancer immunotherapy. Clinical studies in the past have demonstrated the safety of DNA vaccination in humans, showing minimal adverse reactions and the ability to induce a wide range of specific immune responses, however, the therapeutic effects have been minimal which has impeded the progress of this vaccine technology. The primary reason for low immunogenicity of DNA vaccines in humans is due to inherent immunosuppressive properties of the tumor microenvironment, including tumor epitope mutation, T cell exhaustion, antigen tolerance, infiltration of immunosuppressive cells and tumor-associated macrophages that produce immune-suppressing microenvironments (e.g., regulatory/suppressive cytokines) [130]. There are several clinical studies in progress and currently recruiting that focus on cancer DNA vaccines [131]. However, as mentioned previously, the majority of DNA vaccines that have progressed to clinical trials are delivered as naked DNA or through the use of microparticles, as opposed to nanomaterial-based delivery systems. For example, gold microparticles were used to deliver a DNA vaccine encoding the NY-ESO-1 antigen, which is a common biomarker associated with many different forms of cancer [132]. The study showed that the DNA vaccine could induce anti-tumor cell-mediated immune responses, showing it as a potential candidate for cancer immunotherapy. In a more recent clinical trial study, a cationic liposomal adjuvant, JVRS-100, was used to deliver DNA as immunotherapy against leukemia [133]. However, the results have yet to be published for this clinical trial.

## 6. Conclusions and Future Perspectives

Despite the optimistic advancements in the past decade of vaccine research, there are still no commercially available vaccines or effective treatments for many diseases such as cancer, HIV, dengue, zika, and chikungunya. DNA vaccines pose as promising candidates to fill this gap due to the ability to change the vaccine targets simply by changing the protein encoded within the DNA delivered. The main challenge associated with DNA vaccines is generating therapeutically relevant levels of exogenous protein expression within APCs in order to stimulate protective humoral and cell-mediated immunity. The two main methods through which this can be achieved is the use of DNA delivery vectors and immune response enhancing adjuvants. Nanomaterials have been widely researched for use as DNA delivery vectors and adjuvants due to their wide range of properties that can be tuned through surface functionalization. Polymer, lipid, metallic, and inorganic materials have a diverse set of properties with unique advantages and weaknesses, all of which have been seminal contributions in the field of DNA vaccine research, resulting in improved efficacy and more favorable safety profiles.

Despite these advancements in nanomaterial-based delivery systems, DNA vaccines have still performed poorly in human clinical trials to date, failing to generate potent immunogenic responses [7,134]. One reason for this is because preclinical studies are typically conducted on small animal models, which often don’t accurately predict the immune response in humans. The differences in the immune systems between animals and humans are vast, and the immune responses and characteristic behavior of the immune system against any specific pathogen vary significantly. Moreover, the small size of common animal models facilitates the induction of stronger cell-mediated and humoral immune responses with lower doses of DNA vaccines. When these same DNA vaccines are then translated to clinical studies, the resulting immune responses are significantly lower than the preclinical results since the design and implementation were not optimized for humans specifically. Although there have been efforts to develop more relevant preclinical models, for instance, genetically engineering rodents to simulate more humanized models, they all fail to accurately recreate fully competent immune systems of humans. The use of larger animal models could help to bridge the gap between preclinical studies and human trials, however, there are ethical policies, logistical issues, and the need for large economic support that pose challenges [135]. Nevertheless, even larger animal models suffer from many of the same limitations as their smaller counterparts, as such it remains a major difficulty in vaccine research. Currently, a universal immunological model that effectively translates preclinical studies to human trials is impractical and not likely to exist in the foreseeable future.

Since one of the major challenges with DNA vaccines is low immunogenic responses, several other factors including the selection of the encoded antigen, combination therapy, and a treatment schedule can be considered to improve their efficacy in clinical trials. Specifically, decisions such as which antigens should be selected to be encoded within the DNA payload to maximize the effectiveness of the vaccine, as well as how many antigens should be encoded, are crucial details which we do not currently have the knowledge to optimize. Knowing which antigens induce more potent and therapeutically relevant immune responses are important criteria that are often not discussed in preclinical studies which is a major detriment to their clinical success. Moreover, it is unknown what combination of antigens maximizes therapeutic efficacy of the vaccine, and whether antigens should be encoded within the same plasmid DNA molecule, or whether it should be encoded in a separate molecule and administered together. Similarly, it is difficult to predict which adjuvants and co-stimulatory molecules should be used to optimize the effects of the DNA vaccines. The rationale behind selecting one combinatorial treatment compared to another is often overlooked in both preclinical and clinical studies, which presumably limits the efficacy of the DNA vaccines in those study settings. To address many of these issues, more investigative research is required to understand the current capabilities of existing technologies, and the hierarchies of effectiveness. This requires more comparative studies, as opposed to most studies that report on novel designs and systems. Specifically for NP-based DNA vaccines, the major challenges that hinder their translation to the clinic are common issues that affect NP-based drug delivery technologies in general [133]. Most significantly, current NP technologies cannot provide high DNA transfection efficiency capable of stimulating robust immunity. Reasons for this include modulating biodistribution, rapid clearance from the body, insufficient protection against premature degradation and inadequate targeted delivery to immune cells within target tissue. In order to improve the viability of NP-based DNA vaccines, increasing the target site accumulation of NPs while simultaneously decreasing off-target site accumulation is required. More research is required to develop surface ligands for NPs that improve stability in circulation, reducing nonspecific interactions with serum proteins as well as preventing rapid clearance by immune cells. Equally important is the need to identify targets and targeting ligands to enable NPs to effectively target APCs and increase transfection efficiency. Related to this, further advancements in NP design are required in order to facilitate the controlled release of DNA payloads within APCs, and the subsequent antigen processing required to induce protective immunity against the target disease. Additional scrutiny should be utilized to investigate disease microenvironments to see the effects of immune-suppressive cell populations and target those immune-checkpoints to eliminate their functionality in order to improve overall immunity by the vaccine system. Despite the need for further advancements prior to the translation of nanoparticle-based delivery systems for DNA vaccines from the lab to the clinic, it is undeniable that the progress in this field within the past decade has been remarkable. We hope that nanodelivery systems will continue to improve, and as a consequence, so will the efficacy of DNA vaccine technologies. We expect both to become more clinically relevant and therapeutically effective in the near future.
